# ICED OUT: integrated cold-chain eliminating disease outreach. Piloting a motorcycle-enabled laboratory transport system combatting visceral leishmaniasis in rural Kenya

**DOI:** 10.3389/fpubh.2025.1623049

**Published:** 2026-01-21

**Authors:** Alex Chang, Mwatela Kitondo, Edward Obwavo, Hellen Nyakundi, Richard Wamai

**Affiliations:** 1 Department of Biology, Northeastern University, College of Science, Boston, MA, United States; 2 African Centre for Community Investment in Health, Chemolingot, Baringo, Kenya; 3 Department of Health Services, Kolowa Model Health Centre, Kolowa, Baringo, Kenya; 4 Department of Cultures, Societies, and Global Studies, Northeastern University, College of Social Sciences and Humanities, Boston, MA, United States

**Keywords:** last-mile delivery, neglected tropical diseases, phase-change material, remote health infrastructure, visceral leishmaniasis

## Abstract

The fight against neglected tropical diseases (NTDs) in sub-Saharan Africa, particularly visceral leishmaniasis (VL) among Kenya's Pokot tribe, exemplifies the need for improved last-mile healthcare delivery systems. The Pokot face high disease burdens due to their pastoral lifestyle, socioeconomic hardship, and comorbidities such as malnutrition and high exposure to communicable diseases. Despite the availability of VL diagnostics and treatments, limited healthcare access in remote Baringo County exacerbates the disease's endemicity. This study describes a motorcycle-based cold chain transport model for VL diagnostics and vaccine transport in East Pokot, Kenya, utilizing phase-change material (PCM)-cooled transport backpacks. A Likert survey of ten key informants from six healthcare facilities identified critical challenges, including laboratory transport costs, interfacility distance, and lack of cold chain services. Three routes connecting these six facilities to more advanced healthcare facilities were established based on identified gaps. Transport schedules were synchronized with village market days to reduce costs and increase accessibility and frequency. Sample collection was further integrated into World Vision and Kenya Red Cross outreach campaigns. Diagnostic care was thereby extended to over 99 Pokot villages during the pilot period. Implementation revealed key insights into barriers to Pokot health-seeking behaviors, including cultural inhibitors and tribal conflict-related challenges. This model demonstrates potential through its linkage of isolated healthcare facilities, creating greater value efficiency and lower barriers for integration into existing community healthcare frameworks. Lessons learned during the pilot phase can guide implementation of similar community-enabled cold chain transport systems in global regions with healthcare fragility.

## 1 Introduction

In the expanding field of research addressing global health inequities in access, last-mile delivery interventions have emerged as an area ripe for innovative approaches. Borrowed from supply chain economics, where it refers to the cost-reducing delivery of products to end customers, last-mile delivery has received particular attention within global health in the post-COVID era (1–3). With renewed focus on marginalized communities—refugees, those living in extreme poverty, and those in off-grid, geographically isolated regions—the COVID-19 pandemic catalyzed the development of sustainable methods for vaccine delivery and disease diagnostics in last-mile populations, as demonstrated by Sierra Leone's initiatives (3, 4).

Applying this mission to the ongoing fight against neglected tropical diseases (NTDs) in sub-Saharan Africa reveals a critical need within the Pokot tribe of Baringo County, Kenya. The ethnic Pokot are demographically characterized by high household densities and an agro-pastoralist lifestyle, which exacerbates exposure to neglected tropical diseases (5). Among the tropical diseases prevalent in the area, visceral leishmaniasis (VL)—the most serious form of leishmaniasis—remains endemic despite efforts by non-governmental and governmental organizations. In addition to cultural pastoralism (which predisposes child herders to its sandfly vector), poor socioeconomic status manifesting in inadequate diets, low educational attainment, and catastrophic health events trap Pokot families suffering from VL in a cycle of medical poverty and healthcare avoidance (6, 7). A synergistic relationship between comorbid malnutrition, HIV, and VL further exacerbates poor clinical responses to therapeutics and overall health outcomes due to their similar physiological manifestations (7).

An approved vaccine against VL does not currently exist, and diagnostic testing is severely limited by unforgiving terrain and long distances between villages and healthcare facilities. Serodiagnostics and splenic aspirate biopsies require patients to be seen at Level IV healthcare facilities, mandating days of travel without guaranteed treatment. Studies performed in East Africa reveal that access to facilities capable of treatment and diagnosis, costs associated with transport and hospital stays, and lack of disease surveillance are primary factors contributing to endemicity (8, 9). With diagnostics limited to facilities with adequate laboratory capacity and staffing, Pokot individuals suffering from VL and its comorbidities have been restricted to a handful of healthcare facilities scattered across an area exceeding nine thousand square kilometers.

In the continued fight against NTDs in sub-Saharan Africa, novel approaches to cold chain transport must be initiated with community integration in mind. Promising strategies, including UNICEF's non-traditional drone-based laboratory transport corridor in Malawi, have found success in both rural cultural acceptance and addressing community health needs (10). The current study reflects on a pilot model implemented for effective, community-based cold chain transport of diagnostic samples and vaccines for treating visceral leishmaniasis and its comorbidities in East Pokot, Kenya. Utilizing transport backpacks cooled to laboratory-grade temperatures through novel phase-change materials (PCMs) (11), we implemented a three-route local motorcycle transport system to facilitate collection and delivery of laboratory samples for diagnosing VL and its comorbidities, as well as vaccines. The following analysis documents the essential strategy-building and community engagement processes for establishing the ICED OUT Program, which stands for “Integrated Cold-Chain Eliminating Disease OUTreach.” This effort augments the functions of community facilities located in unreached regions of East Pokot, generating insights for culturally sensitive implementation of healthcare infrastructure within a pastoralist, tribal society.

## 2 Materials and methods

### 2.1 Study setting and rationale

This study was conducted in East Pokot, Baringo County, Kenya, among the ethnic Pokot population. Though sparsely distributed, many Level II healthcare facilities (dispensaries) exist that service rural villages and have staff trained to collect human samples and administer vaccinations (12). Compared to Level IV health facilities that provide advanced surgical and laboratory diagnostic procedures at the sub-county level, Level II facilities can only perform minimally invasive laboratory diagnostics requiring little equipment, often referring patients to advanced facilities. The primary issues affecting facility capacity is lack of consistent power and inadequate staffing, compromising refrigeration and laboratory testing.

While existing medical transportation systems are in place, we noted many inefficiencies and opportunities for vector transmission. When suffering from snakebites, trachoma, or other medical emergencies, patients often call upon “Boda-Boda” motorcyclists or all-terrain ambulances to transport them to higher-tier healthcare facilities. These transports are summoned via cell phone and transfer patients from rural villages to Chemolingot Sub-County Hospital (CSCH), a Level IV facility (12). Each transport incurs high per-patient costs, compounded by expenses associated with overnight stays, food, and family caretaker burden; route inefficiency often dissuades patients from seeking care. With undifferentiated illnesses of unknown transmissibility, transport may also expose motorcycle and ambulance drivers to diseases, as they often lack adequate personal protective equipment (PPE).

Our intervention was therefore two-fold. First, our initiative introduced salt hydride PCM-cooled backpacks to transport vaccines and laboratory samples at laboratory-grade temperatures of 3 °C–8 °C. These serve as critical replacements for the smaller, makeshift cooler boxes currently used by local vaccination campaigns and infrequent patient sample transport, which fail to maintain such temperatures and risk the efficacy of vaccines and samples. Second, our program established three new routes connecting multiple isolated Level II facilities to CSCH, creating new Boda Boda courier transport paths that did not previously exist. Although Level II facilities rarely have laboratory capacity for analyzing and storing samples, sample collection often falls within the scope of the healthcare providers managing these facilities. With established linkage routes between Level II facilities and CSCH, healthcare providers within these facilities can collect blood samples from surrounding villages for transport at safe temperatures to CSCH. This essentially extends CSCH's laboratory services to hard-to-reach villages served by Level II facilities, allowing increased accessibility to DAT and RDT blood testing for VL while limiting patient cost and contact.

### 2.2 Needs assessment

From September to October 2023, an initial laboratory assessment was conducted to identify gaps in the existing healthcare system and potential healthcare facilities to incorporate into our new laboratory transport service. The assessment was quantified using a Likert scale questionnaire distributed to key informants within isolated healthcare facilities, primarily surveying nurses and laboratory technicians with frontline experience in sample collection for VL. Healthcare facilities were selected based on their access to a power source, ability to provide medical-grade refrigeration, current involvement in laboratory referrals to Chemolingot Sub-County Hospital (CSCH), and participation in NGO-supported vaccine outreach campaigns.

Questions in the laboratory sample transport survey were categorized as follows: “Timely Delivery and Patient Wellbeing,” “Sample and Transport Quality,” “Cost Accessibility,” “Staff Quality (Staffing, Training, Sample and Patient Protection),” “Geographic Accessibility,” and “Overall Satisfaction.” These categories featured pointed statements about current service delivery, including “Lab samples were consistently delivered within the agreed-upon timeframe” and “Lab samples generally arrived in good condition (well-refrigerated, uncontaminated, etc.).” Rated on a scale of “1–5” with “1” representing “Strongly Disagree” and “5” representing “Strongly Agree,” opinions were quantified with higher scores denoting timely, affordable, and well-preserved transports, while lower scores indicated problems in these qualities.

Vaccine outreach capacities were similarly divided as follows: “Stocking and Capacity,” “Vaccine Efficacy and Quality,” “Cost Accessibility,” “Travel Accessibility,” and “Overall Satisfaction.” The scoring followed the same conventions as the sample transport survey, and score ranges were delineated by median and quartile. These questions covered barriers and efficacy, including questions such as “The mobile vaccine storage during outreach campaigns is adequately cooled and reliable for preserving vaccine viability” and “Geographical barriers (distance, rough terrain, communication, etc.) do not limit the impact of vaccine outreach campaigns.”

The questionnaires were administered in person with minimal intervention unless translation was required. After collecting all completed questionnaires, respondent scores were calculated, scaled, and characterized using predetermined score ranges. Additionally, challenges noted in open-ended questions were categorized and sorted by facility. These analyses were performed in Microsoft Excel. The full questionnaire and analysis can be found in the published dataset.

### 2.3 Phase-change material (PCM) technology selection

Phase-change materials (PCMs) offer an attractive option for thermal energy storage (TES) because they store latent heat. Their effectiveness depends mainly on melting point, heat capacity, and thermal conductivity, which determine charging/discharging rates and temperature control ranges. Organic PCMs include paraffin and non-paraffin compounds, while inorganic types encompass metal alloys, salt hydrides, and molten salts (13). Because performance relies on balancing these properties, an optimal PCM must store and release heat with minimal temperature fluctuation—salt hydrides show strong, cost-effective potential.

Our choice of PCM was governed by facility access to medical-grade refrigeration and the need for sample transports to withstand extreme heat conditions in the field. A hydrated salt solution packed into cold-pack style containers was chosen, featuring a phase change temperature of −3 °C, latent heat capacity of 359kJ/kg, and ability to maintain constant temperatures of −3 °C to 8 °C for 8 h. These cold packs were fitted into 20 L backpacks specifically designed for sample transport (Supplementary Figure 1).

### 2.4 Geographic route mapping and facility selection

Mapping potential transport routes required analysis of the geographic dispersion of VL within Tiaty East and West. Due to government infectious disease regulations, all VL test data is recorded by CSCH, including sample collection dates and village location, tests performed, and results. Data from exactly 1 year prior (November 2022 to November 2023) were analyzed via ArcGIS Pro and heat-mapped against regional healthcare facilities and their laboratory capacities. Based on the epidemiological dispersion of VL prevalence in the area and the capacity of regional facilities, six potential healthcare facilities were chosen as program intervention partners. These Level II facilities featured the largest proportion of VL encounters and referrals to CSCH in the past year and had the minimum infrastructure (working power, on-site refrigeration, and either a nurse or laboratory technician) to participate in this program.

Routes within this area taken by Pokot villagers are mainly unpaved and undocumented in conventional web mapping platforms such as Google Maps. To reconcile this gap, field researchers accompanied by community locals traveled and charted common routes taken by villagers connecting partnered healthcare facilities to CSCH using GPS coordinate mapping. Utilizing these isolated routes, we charted three new routes connecting previously isolated healthcare facilities to other nearby healthcare centers, setting the foundation for our system of synchronized transport days for multiple facilities. These three previously non-existent routes terminate at Chemolingot Sub-County Hospital: the Kolowa–Barpelo Route, the Akwichatis–Riongo Route, and the Loruk Route.

### 2.5 Training of healthcare workers and boda-boda motorcyclists

To ensure appropriate collection and transportation of samples, healthcare personnel from the six selected partner facilities were trained in a one-day training session. Selected healthcare workers, including laboratory technicians, nurses, and clinical officers, were trained on procedures for collecting samples from suspected VL cases and associated comorbidities. Topics included techniques for safe sample collection, preparation, and packaging of blood samples for transport. Participants were also trained in essential recordkeeping practices for later data analysis within this study and maintenance of the cooler backpacks used for sample transport. This training equipped healthcare workers with skills needed to ensure sample integrity from collection to delivery, thereby improving the accuracy of diagnoses and effectiveness of subsequent treatments or referrals.

Boda-Boda motorcyclists underwent a similar 1-day training session. Two Boda-Bodas from each of the three routes were trained on safe transportation of suspected VL blood samples and associated comorbidities from collecting facility to diagnosing facility. Training focused on measures to maintain sample viability during transport, as well as safe practices for transport hygiene, biohazard risk mitigation, and accident management.

### 2.6 Community sensitization campaign

An important component of integration within the existing healthcare system was sensitizing affected villages to our program. Based on previous GIS analysis, regions of East Pokot with the greatest VL prevalence were noted. Villagers in this region were often unaware of protocols for VL treatment and appropriate facilities to obtain care. Those acquainted with treatment and diagnostics needed to be informed that travel to CSCH for testing could be replaced by our transport program, allowing sample collection at their nearest Level II facility.

We used the opportunity granted by our sensitization campaign not only to inform villagers of the greater test accessibility now available through our program but also to provide information on disease presentation and treatment protocol for VL and its comorbidities. Given the pastoralism characterizing Pokot lifestyles, livestock sales at village markets sustain Pokot livelihoods. Thus, village market days, during which Pokot villagers from across the region congregate, were chosen as targets for sensitization. We embarked on an extensive sensitization campaign throughout October–November 2023, during which we visited each village marketplace along our transport routes and engaged in conversations with villagers. Healthcare workers at our partner facilities also aided by sharing information about our transport program with patients, capitalizing on existing trust between patients and healthcare workers to promote our program.

### 2.7 Post-interventional analysis

Health care facilities participating in both laboratory transports and vaccination campaigns were provided logbooks to document each instance of usage. The sample transport logbook from each site recorded laboratory essentials including culture type, tests performed, transport time and temperature, and the dates results were communicated to patients. Cost-benefit analysis of the lab sample transport program compared registered program costs with predicted patient expenses, accounting for transport, testing, and cost of living. Vaccination campaigns documented completed vaccinations and tracked hourly internal backpack temperatures observed throughout the campaign. Data following the interventional period was managed in Microsoft Excel while analyses and visualizations were performed by React, a JavaScript library.

### 2.8 Ethical approval

Ethical approval was obtained from Northeastern University IRB #11-10-18, University of Nairobi Ref UON/CHS/SPH/7/1, Kenyatta National Hospital #P422/10/2011, and Ministry of Public Health and Sanitation, Kenya Ref No. PHS/ADM/2/24. Informed consent was subsequently acquired from all healthcare personnel participants.

## 3 Results

### 3.1 Needs assessment survey results

Ten respondents were sampled from six healthcare facilities in the area. Of these respondents, five were from Chemolingot Sub-County Hospital (two nurses and three laboratory technicians), two nurses from Loruk Health Center and Riongo Dispensary, and three additional laboratory technicians from Kositei Dispensary, Kolowa Model Health Center, and Barpelo Dispensary. Seven respondents completed the Laboratory Sample Collection Survey, and seven completed the Vaccine Campaign Survey. Scores of respondents from the same healthcare facility were averaged to account for discordant responses, though none were ultimately noted. Averaging allowed these healthcare centers to be represented by single data points. Although a larger sample size was desired, our sample was limited due to severe understaffing at regional Level II facilities. Respondents represented all nurses and laboratory technicians available at the chosen facilities. The discrepancy between sample size and participants completing each survey resulted from nurses and laboratory technicians stating they lacked sufficient experience in the other role, as nurses assisted with vaccination campaigns while laboratory technicians managed sample handling and analysis.

A preliminary survey on challenges faced by laboratory services among the selected facilities revealed that the greatest barriers pertained to travel and adequate transportation mediums. Five of seven respondents reported struggles with laboratory transport costs, interfacility distance, and lack of available Boda Bodas. Four of seven respondents endorsed poor road infrastructure and lack of refrigerated transport. Notably, Loruk Health Center and Kositei Dispensary described communication barriers, citing inadequate internet and cellular data connectivity as inhibitors to organizing laboratory sample transport. Although the diversity of challenges described for vaccine outreach campaigns was far less than for laboratory transports, three of seven respondents still cited outreach inconsistency, and two of seven noted vaccine stock shortages as significant barriers to comprehensive immunization campaigns.

Within the Laboratory Sample Collection Survey, a trend emerged between distance from an advanced health facility (i.e., Chemolingot Sub-County Hospital) and capacity for adequate laboratory services (Supplementary Table 1). In almost all categories, Chemolingot Sub-County Hospital reported the highest average Likert scores (indicating better laboratory collection infrastructure and delivery), second only to Kositei Dispensary, which is the closest to Chemolingot Sub-County Hospital among the other facilities. Representing the more distant facilities, Loruk Health Center and Riongo Dispensary consistently scored among the lowest in most categories. Both facilities reported poor delivery of results, poor sample and transport quality, very poor staff quality, and very inaccessible geographic accessibility. Among other factors, these results were primarily induced by severe understaffing, distance and inadequate infrastructure hindering their catchment populations from seeking care, and unsatisfactory preservation of transported samples.

Responses in the Vaccine Campaign Survey demonstrated much less diversity, as noted in Supplementary Table 2. All sites reported that vaccine efficacy during outreaches was of very good quality. Despite consistency in outreach campaign scheduling, travel accessibility was still rated as inaccessible at all facilities, as all agreed that geographic barriers limit the potential impacts of current vaccine campaigns. The greatest differences observed in responses pertained to vaccine stock and cost accessibility. Barpelo and Riongo dispensaries reported that vaccine stock shortages and restock delays inhibit outreach potential. Both sites also projected that their respective systems would be ill-equipped to manage unexpected vaccine demand surges and outbreaks. Additionally, cost appeared to make vaccine outreaches very inaccessible in both systems, though neither site detailed a reason for this response.

### 3.2 Implementation outcomes

**Figure 1:**
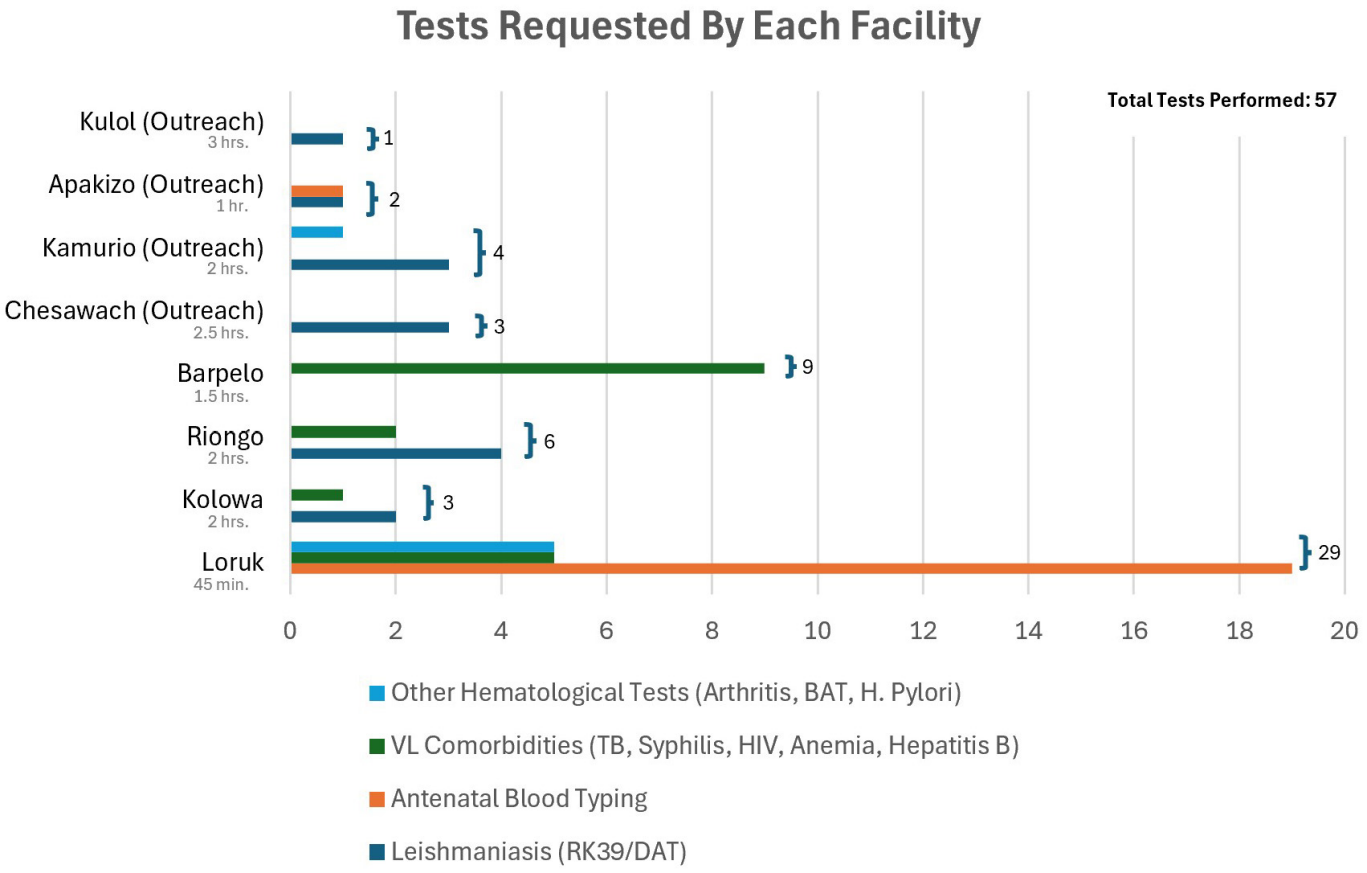
Sending facilities and their respective tests requested to be performed at Chemolingot Sub-County Hospital (CSCH). Total tests are summed by facility, and travel time between facility and CSCH are noted.

This pilot model was implemented for 5 months (October 2023–March 2024) following the village sensitization campaign. Two Boda Boda riders were assigned to each of three routes, employing six motorcyclists in total. Laboratory specimens were transported biweekly, while vaccine consignments followed Kenya Red Cross and World Vision schedules. Diagnostic care was effectively extended to an area covering more than 99 Pokot villages. Over the pilot period, riders successfully completed eleven transports to serve 56 patients from four peripheral health facilities and four village outreaches. Transport time varied by sending facility, with the shortest (and most frequent) 45-min trip occurring from Loruk, and the longest 3-h transport arriving from an outreach campaign in Kulol. Of the 57 tests performed, 14 tested for VL and 17 tested for its comorbidities, including TB, syphilis, HIV, anemia, and hepatitis-B (Figure 1). Unexpectedly, the sample transport program was also heavily utilized in antenatal care, with 19 mothers from Loruk region requesting antenatal blood grouping and rhesus typing.

**Figure 2:**
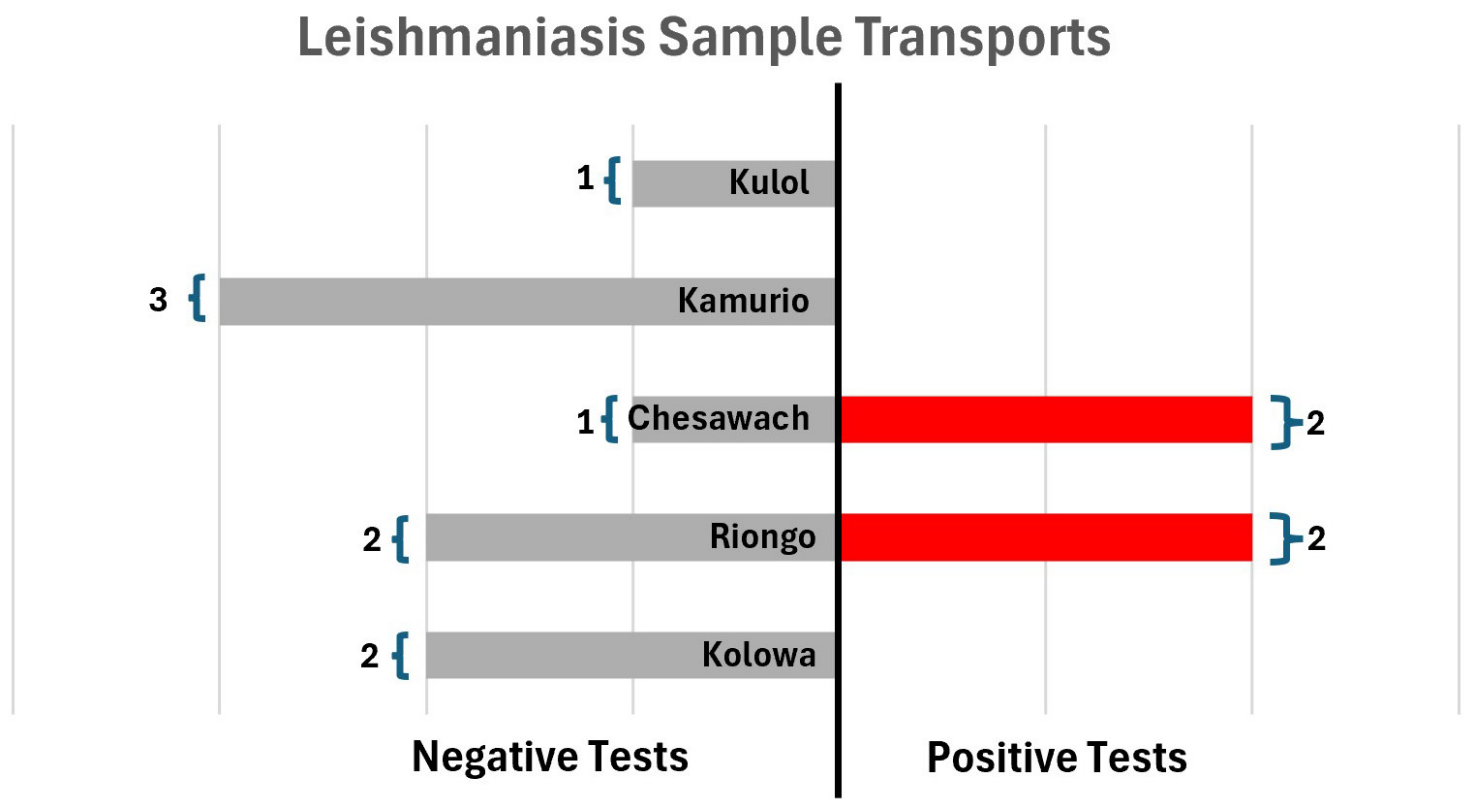
VL samples categorized by test result and sending facility.

**Figure 3:**
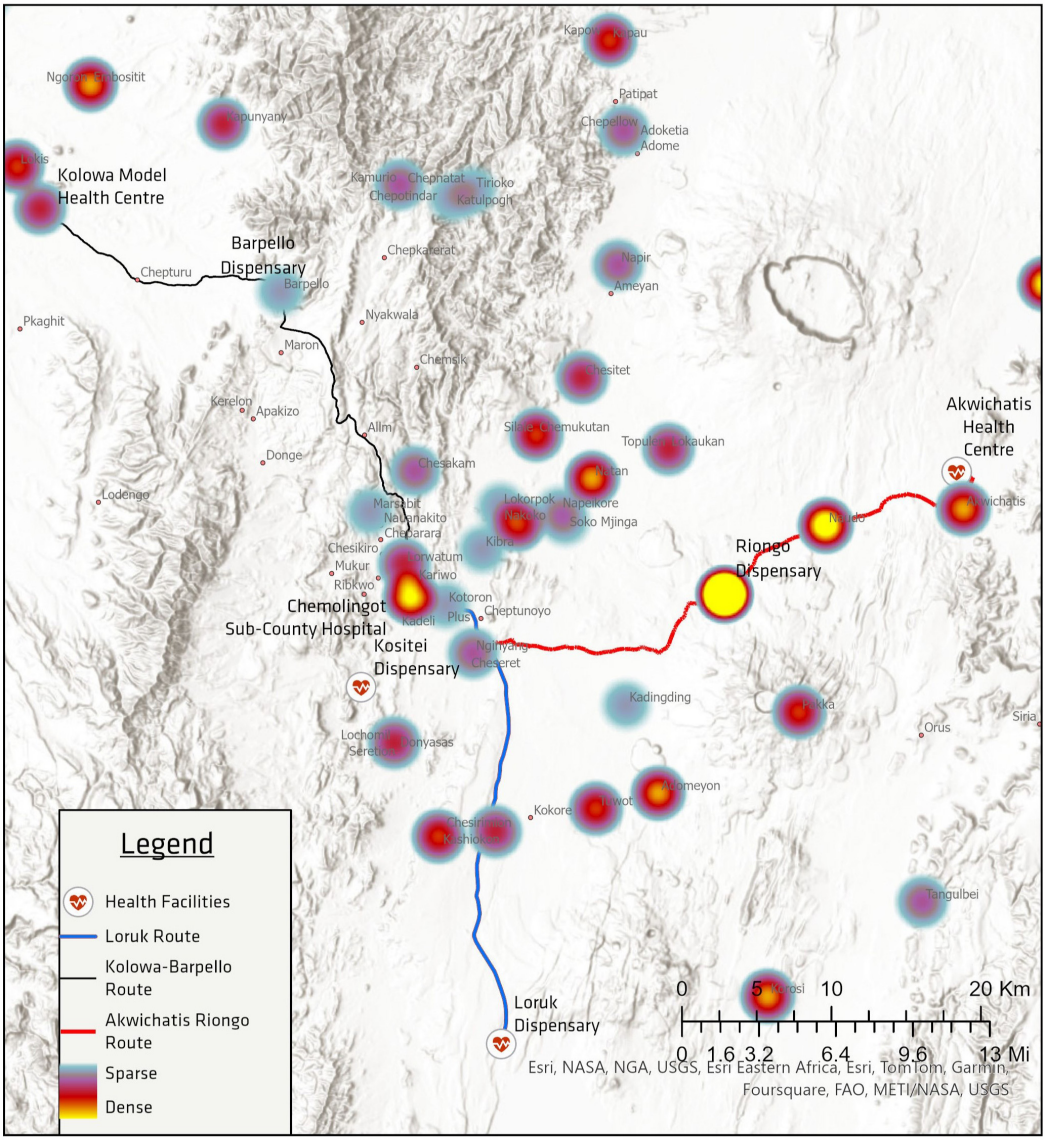
Heat map of VL cases from November 2022–November 2023 overlayed upon implemented laboratory transport routes. ‘Sparse' and ‘Dense' refer to disease prevalence. Three routes (as denoted by the bolded lines) were designed based on disease prevalence among villages in East and West Pokot to connect level II health facilities with the only level IV health facility in the region, Chemolingot Sub-County Hospital.

Visceral leishmaniasis was successfully detected in four out of fourteen patients exhibiting consistent symptoms, and three out of four positive patients were contacted within 1 day of sample collection (Figure 2). More broadly, test results for all samples were, on average, communicated in about half-days' time (*M* = 0.65 days, SD = 1.55 days), with 36/56 patients contacted on the same day. Notably, the geographic distribution of facilities requesting VL diagnostics seem to align with the VL hotspots of the preceding year as seen in Figure 3, specifically in Riongo, Kolowa, and the interior regions of East Pokot.

**Figure 4:**
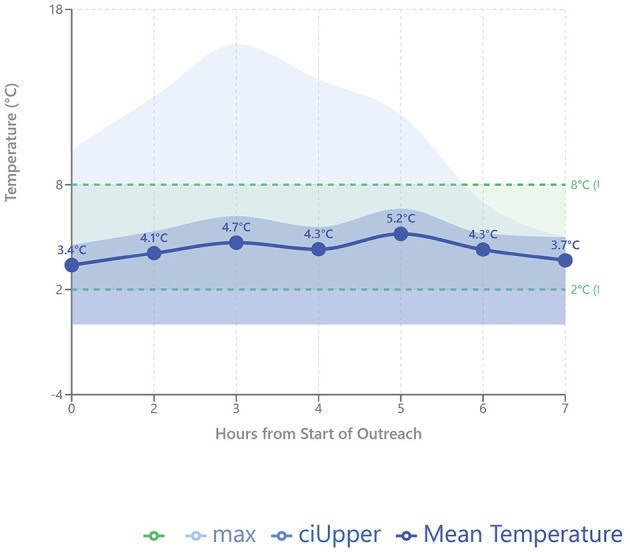
Mean temperature trajectory (*n* = 8–18 campaigns per timepoint) with 95% confidence intervals. Shaded area indicates recommended cold chain temperature range (2 °C−8 °C).

Laboratory sample transports were received at an average temperature of 3.9 °C (*M* = 3.85 °C, SD = 1.68 °C), though a more complete gauge on temperature can be observed through the documented vaccination campaigns. Eighteen vaccination outreaches were successfully completed within the study period. Carried on these campaigns were BCG, Rotavirus, OPV, IPV, Pentavalent, TT, yellow fever, and measles vaccines, with an average of eight patients vaccinated per outreach. Within the months of January and February, CSCH also embarked on an HPV vaccination series, providing services to five underserved Pokot primary schools and averaging forty children vaccinated per outreach. Temperatures were recorded at the beginning of each outreach and tracked hourly until the end of the campaign day, when vaccines were returned to CSCH (Figure 4).

Among 18 campaigns, 15 maintained proper cold chain temperatures throughout the outreach day. Observed within the temperature trajectory was a gradual peak between 3 and 5 h as the storage apparatuses were opened with each vaccine that is administered, followed by a return to baseline as they were transported back to CSCH. The upper 95% CI demonstrates narrow variance, indicating reliable temperature control, and the overall mean temperature of 4.23 °C is well within the ideal range. One campaign to Chesawach represented a clear outlier with a temperature range of 10 °C−16 °C, but this was most likely due to protocol deviations, such as leaving open backpack compartments, which should be addressed with training.

### 3.3 Cost analysis

The program's strengths are most clearly seen through cost analysis. We performed a cost analysis comparing the cost of each program transport with the projected individual transport, living, and laboratory costs that would have been incurred had each patient pursued testing on their own. These costs are defined in Table 1.

**Table 1:** Costs in KSH based on current Boda-Boda rates for transport and laboratory fees at CSCH. Daily living cost encompasses food and accommodation for patients staying overnight in Chemolingot as they await results and treatment.

**Sending facility**	**Travel cost (KSH)**	**Laboratory test**	**Cost per test (KSH)**	**Daily living cost (KSH)**
Kulol	8,000	RF test-arthritis	200	400
Chesawach	8,000	H. Pylori	150	400
Apakizo	4,000	BAT	200	400
Kamurio	2,000	Hemoglobin	150	400
Kolowa	1,500	ANC	200	400
Riongo	1,500	VDRL/viral load - syphilis/HIV	150	400
Barpelo	1,500	Rh factor	150	400
Loruk	1,200	Hepatitis-B	150	400
–	–	Blood grouping	100	–
–	–	RK39/DAT leishmaniasis	Free	–
–	–	TB	Free	–

Prior to program initiation, each client was required to travel to CSCH, often with a caregiver, and spend at least one overnight awaiting test results. Accordingly, we calculated the projected costs as a summation of transport costs to CSCH, laboratory costs, and daily living. With the introduction of our program, the greatest reductions occur in transport and cost of living, as groups of patient samples collected at one facility only require one Boda Boda transport fee, and patients are saved both travel and accommodation costs as they can quarantine within their villages. The 56 samples transported during the study period cost 43,300 KSH, which was mostly absorbed by program grants and the collective of partnering healthcare facilities. Compared to the projected 135,900 KSH or more that would have been spent by these patients without our Boda-Boda program, the cost savings of 92,600 KSH represents the conservation of almost five Pokot household incomes, which average 19,000 KSH annually (14).

## 4 Discussion

### 4.1 Principal findings

Our low-threshold laboratory transport system demonstrates clear promise in both cost efficiency and improved testing access. Its strength lies in mobilizing local resources—leveraging existing health facilities and motorcyclists to collaboratively expand regional diagnostic capacity. The needs assessment revealed specific gaps in refrigeration, road accessibility, and cost efficiency with the current model of sample and vaccine transport. The subsequent design of routes linking and coordinating multiple healthcare centers in place of their existing isolated model, combined with replacement of makeshift cold boxes with PCM refrigerant backpacks, directly addressed these needs. By having one Boda Boda visit multiple connected healthcare centers, costs for individual facilities decreased drastically, as Boda Boda transports could be negotiated at lower rates with greater transport frequency.

Operationally, the evidence gained from the 56 sample transports and 18 vaccination campaigns supports our proposed efficacy and cost-effectiveness. Narrow confidence intervals in the average temperatures maintained throughout vaccine campaigns, combined with the lack of denatured samples, demonstrates the reliability of PCMs, even in the hot, arid Pokot environment over long distances. And while cost-effectiveness is significant with our community cost reduction of 92,600 KSH, greater still are the gains in our patients' treatment timeliness and relief of burden. From our field notes, villagers noted unsustainable travel times, where walking from Kolowa or Loruk to CSCH would take 8–12 h, and traveling from interior villages like Kulol and Chesawach could take multiple days. Seeking care represents an immense undertaking; a study from the neighboring Turkana County estimated that the mean delay time since symptom onset to seeking care for VL was 46.9 days due to accessibility issues (15). As Mbui et al. support a decentralized facility model for greater diagnostic and treatment accessibility, our pilot aligns with this approach, enabling peripheral facilities through a cold chain connection with CSCH. Although patient follow-up was not documented in this pilot, the same-day turnaround for communicating results to patients in this study offers a potential bridge across geographic barriers. Anecdotally, a mother in Loruk discovered an Rh type that risked her fetus' health—care she stated she would not have pursued at CSCH without the sample transport service. We are hopeful that experiences with swift result turnarounds through our system gradually dismantles delays in seeking care for the Pokot.

Yet still understated is the potential for scale, not only in geographic coverage, but also in scope of practice. Effectively, our pilot connected patients to the appropriate facility without need for referral, and limited travel for patients who did not have VL. The ability of sample analysis and diagnosis without the requirement of patient transport opens opportunities for zoonotic disease surveillance while maintaining quarantine, and the usage of samples directly from the field to develop future treatments.

### 4.2 Cultural considerations and health-seeking behaviors

Understanding Pokot perspectives on health is essential for effective intervention. Among the Pokot, well-being encompasses biological, interpersonal, and spiritual dimensions. While biological manifestations align with Western medicine, illness is often attributed to moral transgressions (ngoku), condemnation by God (Tororot), or malevolent spirits (onyetei) (16). These beliefs subsume the biological plane, making spiritualism a dominant form of care; traditional healers are usually consulted first, and biomedical services sought only when conditions become severe. Misconceptions regarding blood collection and fears of witchcraft became topics to reconcile during the community sensitization campaign undertaken by this pilot and were crucial for local leaders to endorse our program.

Culturally responsive approaches must respect local norms. Experiences from Malawi's drone-enabled sample delivery system highlight how anxieties about witchcraft, privacy, and security can undermine innovation, underscoring the need for culturally attuned education (10). In future expansions, partnering with village elders should be emphasized to foster community acceptance and normalize new delivery models and more diverse understandings of medicine.

Cultural dynamics further interact with insecurity, constraining access to care. Pastoralism predisposes children grazing livestock to visceral leishmaniasis, while raids and cattle rustling—intensified by small arms, competition for grazing land, and dowry obligations—generate chronic instability along borders with Elgeyo Marakwet and South Baringo (17, 18). During the pilot, such tensions forced temporary clinic closures and evacuation of non-local staff, discouraging Boda Boda transporters and limiting villagers already facing cultural and geographic barriers. Collaboration with neutral organizations (e.g., Red Cross, World Vision) may mitigate these risks.

### 4.3 Future implications for policy and practice

Despite these challenges, there are several key implications that emerge from this pilot study. First, the synchronization of transport schedules with village market days demonstrates the importance of aligning health interventions with existing community patterns. This approach not only reduces costs but also increases cultural acceptability and participation. Second, the training of both healthcare workers and Boda Boda motorcyclists emphasizes the need for capacity building across all stakeholders in last-mile delivery systems. By equipping local personnel with appropriate skills and knowledge, the program ensures sustainability beyond the pilot phase. Third, the integration with existing NGO outreach campaigns (World Vision and Kenya Red Cross) highlights the value of coordinating efforts across organizations rather than creating parallel systems. This collaborative approach maximizes resource efficiency and reduces duplication. While much remains to be learned as the model advances toward scale, we hope that the cultural insights and implementation strategies developed through this pilot can inform similar efforts to strengthen laboratory access and preventive services in other pastoralist and resource-limited settings.

## 5 Limitations

Of course, our study was not without limitations. Firstly, the sample size of the Likert Scale survey was limited due to the severe staff shortage shared across all healthcare facilities in the area. Because this program was enacted in an austere medical environment, there was lack of freedom to have a rigorous sample selection process, and the survey was provided to all healthcare staff available. Comparisons to transport operations prior to our program was also unable to be completed, as the few operations that existed were majorly undocumented, and did not describe critical aspects as cost, temperature management, and samples analyzed. Additional environmental factors posed challenges to the implementation of this program. During the study period, large-scale flooding occurred throughout Western Kenya, halting transport operations for weeks on end due to hazardous road conditions and the destruction of bridge infrastructure. Ethnic conflict between the Pokot and Turkana tribes in the form of cattle rustling were also threats to the safety of the Boda Boda and hospital personnel, resulting in several weeks of transport stoppages. The evaluation of this pilot model would benefit from a longer, consistent study period with a more in-depth cost analysis and a documentation of village integration that was not possible with the granted study period.

## Data Availability

The datasets presented in this study can be found in online repositories. The names of the repository/repositories and accession number(s) can be found below: doi: 10.6084/m9.figshare.28903004.
