# Supplementary figures and images for: ICED OUT: integrated cold-chain eliminating disease outreach. Piloting a motorcycle-enabled laboratory transport system combatting visceral leishmaniasis in rural Kenya
Source: Front Public Health. 2026 Jan 21;13:1623049. doi: 10.3389/fpubh.2025.1623049 (PMC12868197; doi:10.3389/fpubh.2025.1623049)

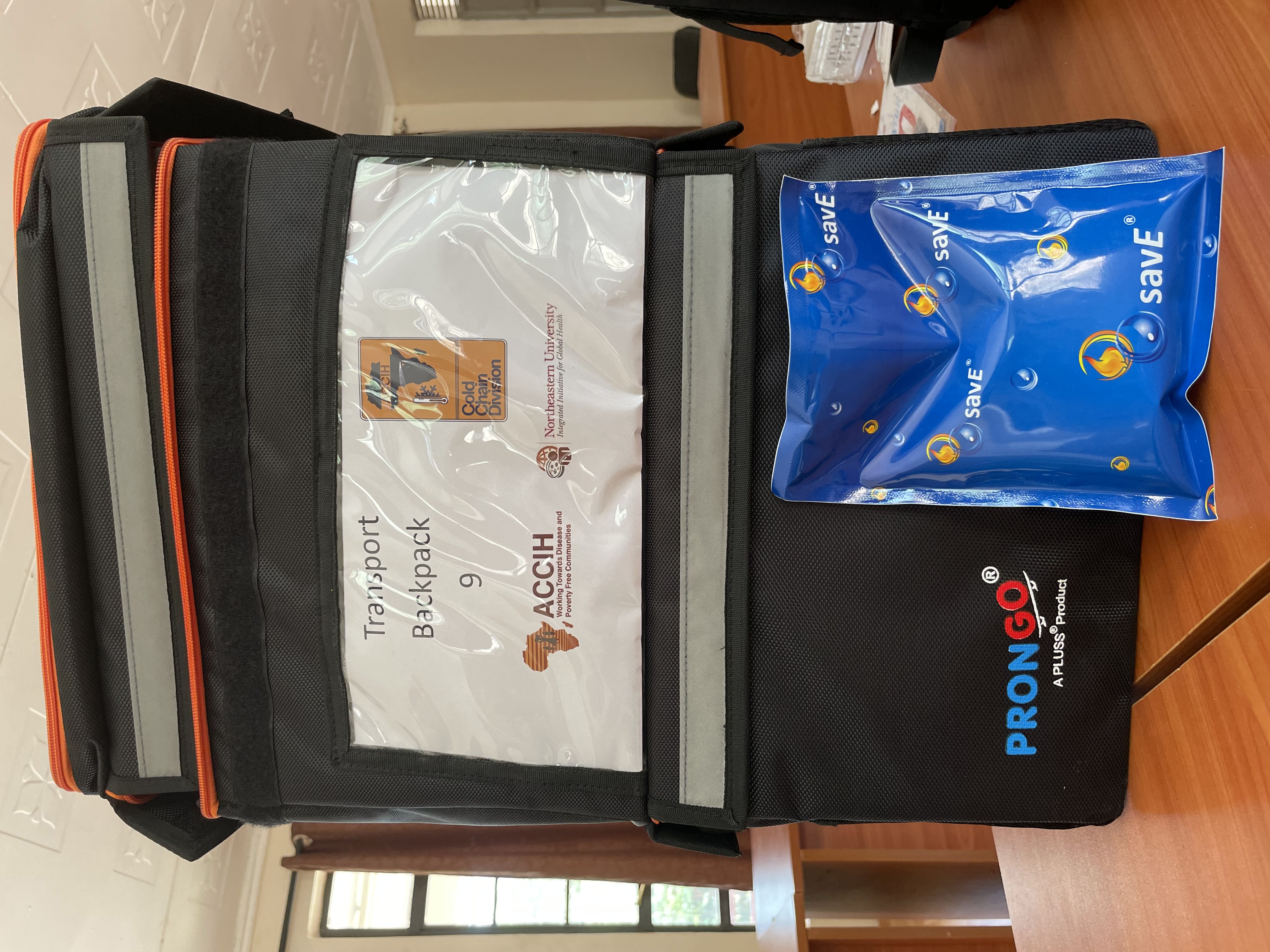

Supplement: Supplementary file 1 [file Image_1.jpeg]

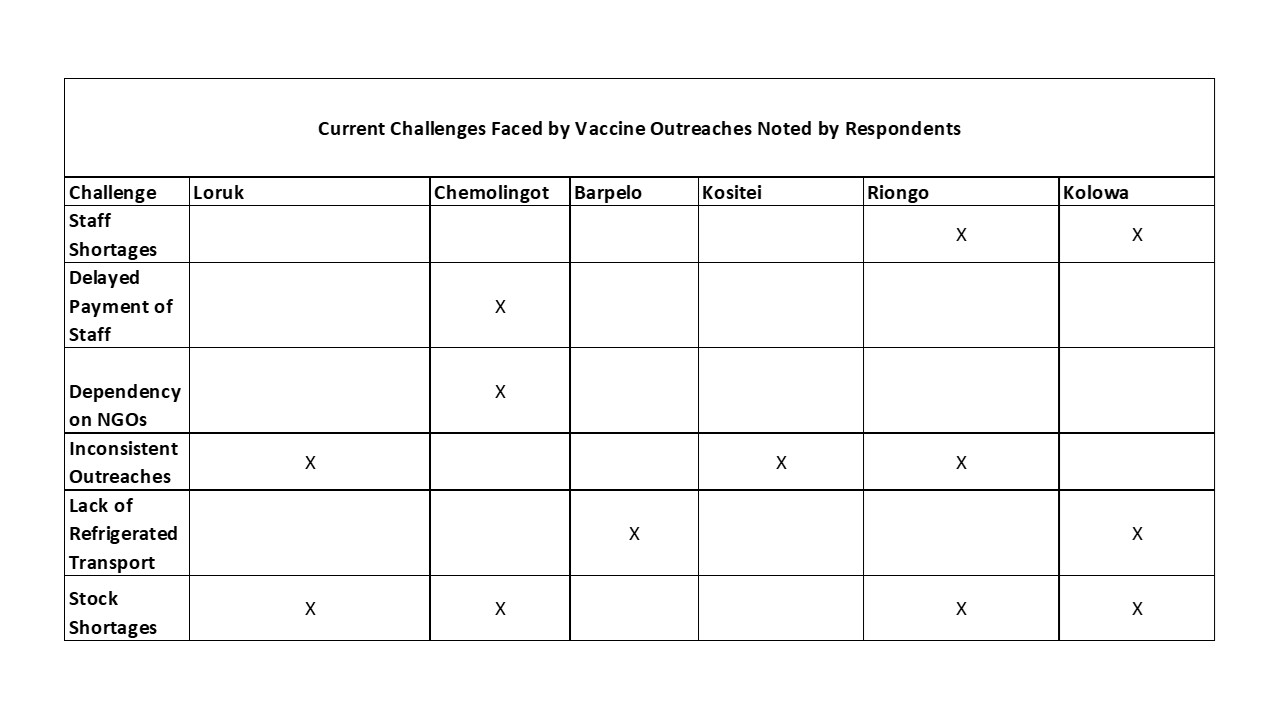

Supplement: Supplementary file 2 [file Image_2.jpeg]

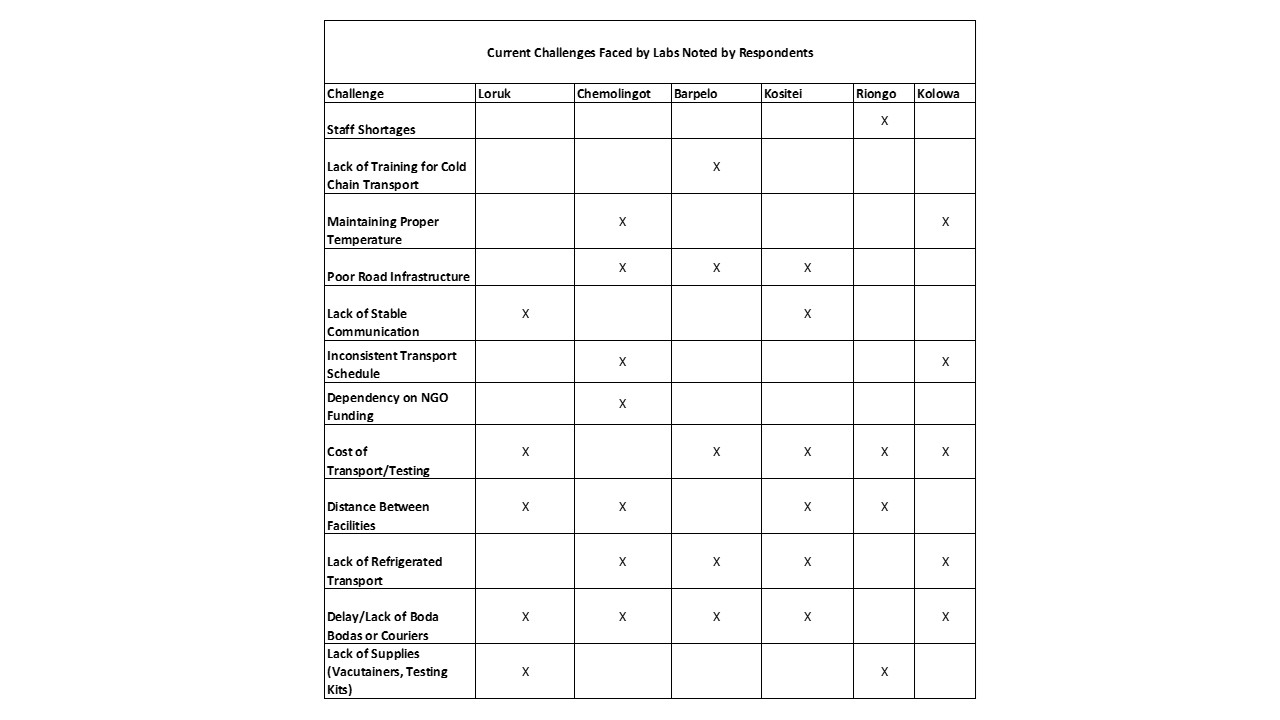

Supplement: Supplementary file 3 [file Image_3.jpeg]
